# Consensus‐Dominant Futures in Nursing: A Co‐Citation and Co‐Word Science‐Mapping Analysis of Future‐Framed Scholarship (1990–2025)

**DOI:** 10.1155/nrp/1839511

**Published:** 2026-08-13

**Authors:** Alex S. Borromeo, Estelle Rose M. Lerma, David S. Bote, Marlyn M. Cabungcal, Walton Wider

**Affiliations:** ^1^ College of Nursing, Bulacan State University, Malolos, Bulacan, Philippines; ^2^ Faculty of Business and Communications, INTI International University, Nilai, Malaysia, newinti.edu.my

**Keywords:** bibliometric analysis, futures thinking, health policy, nursing leadership, scenario planning, strategic foresight

## Abstract

**Background:**

Futures thinking considers multiple possible, preferred, and undesirable futures; strategic foresight applies this orientation to planning. Nursing leaders face workforce instability, digital health adoption, and recurrent disruptions that demand anticipatory action.

**Purpose:**

To map how future‐framed approaches appear in nursing‐indexed journal literature (1990–2025) and identify leadership, education, and policy gaps.

**Methods:**

English‐language Web of Science Core Collection journal articles and reviews (searched 08 Nov 2025) were screened for a substantive future‐framed purpose in nursing. Co‐citation analysis mapped frequently co‐cited references, and co‐word analysis mapped high‐frequency author‐keyword patterns.

**Findings:**

The Web of Science search generated *n* = 4478 raw imported records. After sequential metadata‐based screening, *n* = 3137 records were retained as the final verified eligible corpus. Co‐citation and co‐word analyses identified four citation‐based clusters and four author‐keyword themes within the screened corpus. Within the retrieved corpus, co‐citation patterns were consensus‐dominant, anchored in Delphi guidance, consensus measurement, and reporting standards, alongside methodological and leadership/education reform texts. Co‐word themes clustered around clinical risk, outcomes, long‐term care, and measurement; nursing workforce, quality, leadership, patient safety, and work environment; Delphi/consensus‐based nursing management and guideline development; and nursing education, implementation, attitudes, and learning/program development.

**Conclusions:**

Within the indexed journal record, future‐framed nursing scholarship is growing but is most often operationalized through consensus‐oriented pathways; explicit foresight tools such as scanning, scenarios, backcasting, and futures literacy remain comparatively sparse in titles, abstracts, and keywords.

**Highlights**
• Screened 4478 raw Web of Science records and mapped a verified corpus of 3137 future‐framed nursing records (1990–2025).
• Co‐citation patterns are consensus‐dominant (Delphi guidance/standards).
• Co‐word themes center clinical risk and measurement, workforce quality and leadership, Delphi/consensus‐based guideline development, and nursing education/implementation.
• Explicit foresight tools (scanning, scenarios, and backcasting) are sparse in metadata.
• Identifies publication‐visible gaps in explicit foresight tools within nursing‐indexed journal metadata.

## 1. Introduction

Nursing is increasingly practiced in a world marked by demographic aging, rising chronic disease, rapid technological change, and recurrent public health emergencies. These pressures intensify long‐standing concerns about quality and safety, as organizational structures and care processes continue to shape patient outcomes [1]. Large multicountry studies have shown that nurse staffing, educational preparation, and work environments are associated with mortality, adverse events, burnout, and job dissatisfaction, positioning nurses as central to the resilience and future performance of health systems [2, 3]. Policy and professional frameworks—including The Future of Nursing: Leading Change, Advancing Health, the AACN Essentials, and The Future of Nursing 2020–2030: Charting a Path to Achieve Health Equity—similarly call for expanded nursing roles in leadership, systems transformation, innovation, and health equity [4–6].

Despite this mandate, many professional and educational frameworks offer limited operational guidance on how nursing leaders and educators can translate alternative futures into workforce, education, and policy decisions. In this review, futures thinking is treated as a plural‐futures orientation, strategic foresight as an applied planning practice, and Delphi‐related or consensus methods as convergence approaches that may support anticipatory planning when explicitly framed toward future priorities, competencies, standards, or service directions [7–11]. Delphi is therefore not treated as a proxy for futures thinking but as one publication‐visible pathway through which future‐framed concerns may be translated into nursing priorities, standards, or guidance [11–14].

The term “nursing foresight” is used here as a pragmatic organizing label for journal‐indexed, future‐framed applied inquiry that is visible and retrievable through bibliographic metadata [7, 8, 15, 16]. The corpus should therefore be understood as a map of retrievable future‐framed nursing scholarship rather than an exhaustive account of all work on nursing futures. It includes studies that explicitly use futures or foresight constructs, as well as prospective consensus‐based studies only when authors frame them as future planning, anticipatory role or competency development, forward‐looking priority setting, or future‐oriented standards/guidance in nursing.

Bibliometric science mapping is used to describe how future‐framed nursing scholarship is cited, indexed, and clustered in the journal record. Co‐citation analysis identifies frequently co‐cited references, while co‐word analysis identifies author‐keyword concentrations [15–18]. The aim is descriptive and diagnostic rather than predictive or prescriptive [16, 18].

This study therefore maps future‐framed nursing scholarship from 1990 to 2025 to (1) describe the verified corpus and its publication profile; (2) identify frequently co‐cited references and citation‐based clusters; (3) map author‐keyword concentrations; and (4) identify publication‐visible gaps in the representation of explicit foresight tools.

## 2. Methods

### 2.1. Bibliometric Analysis

Bibliometric analysis is a quantitative research method used to map co‐citation and co‐word patterns in research on futures thinking and strategic foresight in nursing. Bibliometrics quantifies patterns in publications and citations to reveal influential works, actors, and themes [15, 18]. Bibliometric approaches have similarly been used to map publication patterns, influential works, field structures, and thematic evolution across different research domains [19, 20].

To capture both the intellectual structure and thematic evolution of the field, two complementary techniques were used. Co‐citation analysis was used to identify frequently co‐cited references and citation‐based groupings. Co‐word analysis was used to identify frequently co‐occurring author keywords and metadata‐visible thematic concentrations.

### 2.2. Search Strategy and Data Collection

A comprehensive bibliometric search was conducted in the Web of Science Core Collection (SCI‐EXPANDED, SSCI, and ESCI) using Topic Search (TS) fields on 08 November 2025 to identify peer‐reviewed literature on futures thinking and strategic foresight in nursing. This search produced a raw imported dataset of *n* = 4478 records. Because the retrieval strategy was intentionally broad, the imported records were not treated as automatically eligible. Instead, all records underwent sequential metadata‐based screening to verify nursing relevance, future‐framed purpose, document type, language, and source eligibility. Records were retrieved when relevant terms appeared in the title, abstract, or author keywords. The search combined nursing terms with futures or foresight anchor terms and, where relevant, systems or complexity qualifiers used only in conjunction with a futures or foresight anchor. The full search strategy and its justification are reported in Supporting Table S1.

Term selection followed a construct‐to‐proxy logic because future‐framed nursing work is inconsistently labeled in bibliographic metadata. Accordingly, the search emphasized retrievable proxies such as futures thinking, strategic foresight, foresight planning, scenario planning or analysis, horizon or environmental scanning, backcasting, futures literacy, anticipatory governance, visioning, three horizons, the futures triangle, trends, and megatrends. Systems‐thinking terms were treated as contextual qualifiers rather than futures proxies and therefore could not retrieve general systems‐thinking nursing papers on their own. The eligibility treatment of Delphi and other consensus‐based studies is specified below under Eligibility Criteria to avoid repeating the same conceptual caveat across the Methods section.

### 2.3. Operational Boundary for Futures Orientation

Because some retrieval proxies (e.g., systems thinking and complex adaptive systems) are widely used across nursing research without any futures orientation, this review applied an explicit boundary rule to reduce conceptual drift and prevent the corpus from collapsing into “general nursing” literature. Records were treated as futures‐oriented only when at least one futures/foresight anchor term was present in the title, abstract, or author keywords (e.g., strategic foresight, foresight, futures thinking, futures studies, scenario planning/analysis, horizon/environmental scanning, anticipatory governance, futures literacy, backcasting, visioning, three horizons, and futures triangle) and the article’s stated purpose reflected future‐framed inquiry during screening [7, 8]. Broader planning or systems terms (e.g., systems thinking and complex adaptive systems) were therefore treated as contextual qualifiers rather than defining indicators of futures orientation and were retained only when co‐occurring with a futures/foresight anchor and an explicit future‐framed purpose.

### 2.4. Why Structured Methods (Science Mapping) Were Used

This review aims to map frequently co‐cited references and author‐keyword concentrations in future‐framed nursing scholarship at scale. Co‐citation analysis is suited to identifying influential references and citation‐based groupings through patterns of joint citation [15, 18, 21]. Co‐word analysis is suited to identifying frequently occurring themes and metadata‐visible topic groupings through keyword co‐occurrence, reflecting how authors describe and cluster topics in the published record [15–17]. Together, these techniques fit the study’s descriptive, mapping‐oriented aims and make publication‐visible patterns in the field more visible without requiring a priori classification of topics.

An initial exploratory search covering 1970–1989 was also conducted to identify earlier nursing‐focused publications that met the eligibility criteria for foresight, Delphi‐based future planning, horizon scanning, scenario approaches, or related anticipatory methods. No eligible nursing‐focused publications were identified prior to 1990 under the present operational definition and database scope; the timeframe was therefore restricted to 1990–2025 to reflect the modern era when structured consensus methods and futures‐oriented policy agendas became more prominent in nursing and health systems planning.

### 2.5. Eligibility Criteria

Inclusion criteria were (1) English‐language journal articles or reviews indexed in Web of Science; (2) nursing‐relevant records, including nursing as a discipline, workforce, education, leadership, regulation, roles, practice models, or nursing‐led service planning; and (3) substantive futures orientation, operationalized as either

a. explicit use of futures/foresight constructs (e.g., foresight, scenarios, scanning, futures literacy, and anticipatory governance) with an articulated future‐framed purpose or
b. prospective Delphi‐ or consensus‐based studies in which authors explicitly framed the study purpose as future planning, anticipatory role or competency development, forward‐looking priority setting, or future‐oriented standards/guidance in nursing.

Exclusion criteria were (1) editorials, letters, commentaries, notes, conference papers, book chapters, theses, preprints, and other non‐peer‐reviewed outputs; (2) articles lacking a substantive focus on foresight, future planning, or anticipatory thinking in a nursing or healthcare context; and (3) studies using consensus methods solely for present‐focused guideline development without an explicit future‐framed rationale.

Co‐citation and citation‐based mapping require stable, standardized bibliographic metadata and systematically captured reference lists to produce interpretable network metrics [15, 16, 18, 21]. Gray literature (e.g., reports, policy briefs, and national foresight exercises) is often inconsistently indexed and variably cited, which can introduce structural bias in co‐citation networks and reduce reproducibility across runs. For these reasons, the dataset was restricted to English‐language peer‐reviewed journal literature indexed in Web of Science Core Collection, with consistent indexing and reference capture. This design improves bibliometric reproducibility but narrows dataset comprehensiveness, particularly by underrepresenting applied foresight work, policy documents, gray literature, and nursing‐relevant publications indexed outside Web of Science.

This approach ensured that the final dataset was not defined by broad keyword retrieval alone. Records were retained only when they passed both bibliographic eligibility criteria and metadata‐based content screening for nursing relevance and future‐framed purpose. The final screened corpus should therefore be interpreted as a verified, metadata‐supported corpus of future‐framed nursing scholarship, rather than as the unfiltered output of the initial Web of Science search.

### 2.6. Screening Verification and Audit Trail

To strengthen corpus validity and reproducibility, all imported Web of Science records were re‐audited at the record level using a structured Excel screening log. Duplicate checking was performed using the Web of Science accession number (UT). The screening sequence then applied the following criteria: publication period 1990–2025, English language, journal record status, article/review document type, nonretracted status, explicit nursing‐focused evidence, and future/foresight or systems‐context evidence in the available metadata. Nursing‐focus evidence was assessed using metadata fields, including title, source title, author keywords, Keywords Plus, Web of Science category, and research area. Future‐framed evidence was assessed using title, abstract, author keywords, Keywords Plus, and related metadata fields. Each record was assigned pass/fail decisions and, where excluded, an exclusion reason. The complete screening audit trail is provided as Supporting Data File S1.

### 2.7. Network Parameterization and Thresholds

Network construction and visualization were performed using VOSviewer version 1.6.20 with association‐strength normalization following van Eck and Waltman [15]. For the co‐citation analysis, the verified corpus of *n* = 3137 records generated *n* = 97,859 cited references. A minimum citation threshold of *n* = 24 was applied, resulting in *n* = 64 cited references included in the final co‐citation map. This threshold was selected pragmatically through iterative test runs to balance inclusiveness and visual interpretability. The co‐citation network was clustered using a resolution of 0.90 and a minimum cluster size of 1, yielding four clusters consisting of *n* = 20, *n* = 17, *n* = 14, and *n* = 13 cited references.

To improve robustness, a threshold‐proximity sensitivity check was conducted by comparing the final co‐citation threshold of 24 citations with adjacent thresholds of 23 and 25 citations. This check was not intended as formal cluster validation but as a pragmatic assessment of whether the broad intellectual interpretation changed materially across nearby parameter choices. Across these settings, retained cited references ranged from 58 to 70, and the number of clusters ranged from 4 to 5. The dominant intellectual pattern remained stable, with the map continuing to be anchored in Delphi methodology, consensus reporting standards, measurement/reporting guidance, and nursing leadership/education reform texts. The sensitivity summary is provided in Supporting Table S7.

For the co‐word analysis, the verified corpus generated *n* = 9465 author keywords. A minimum occurrence threshold of *n* = 55 was applied, retaining *n* = 61 keywords for visualization. This threshold was selected to preserve the most frequently occurring and thematically central keywords while maintaining interpretability of the map. The co‐word network was clustered using association‐strength normalization, a resolution of 1.00, and a minimum cluster size of 6, yielding four clusters consisting of *n* = 22, *n* = 14, *n* = 13, and *n* = 12 keywords.

A corresponding threshold‐proximity sensitivity check was performed for the co‐word analysis by comparing the final occurrence threshold of 55 with adjacent thresholds of 54 and 56 occurrences. Across these settings, retained keywords ranged from 61 to 63, and the number of clusters remained stable at 4. The broad thematic structure remained substantively consistent, showing concentrations around clinical risk/outcomes/measurement, workforce quality and leadership, Delphi/consensus‐based guideline development, and nursing education/implementation. These checks support the stability of the broad interpretation while acknowledging that exact node inclusion, cluster boundaries, and visual density remain parameter‐sensitive.

## 3. Findings

### 3.1. Descriptive Analysis

The Web of Science search generated *n* = 4478 raw imported records across nine plain‐text export files. Duplicate checking by Web of Science accession number identified no duplicate records. Twenty records published before 1990 were excluded, leaving *n* = 4458 records within the bibliometric period. Source and document‐type screening excluded *n* = 10 non‐journal book/series items and *n* = 3 retracted records, leaving *n* = 4445 records. No non‐English records were identified. Metadata‐based content screening then excluded *n* = 1308 records that lacked sufficient explicit nursing‐focus evidence or future/foresight or systems‐context evidence. The final verified eligible corpus for bibliometric analysis was therefore *n* = 3137.

Separate thresholding procedures were then applied for network visualization. The co‐citation map retained *n* = 64 cited references from *n* = 97,859 cited references at a minimum citation threshold of *n* = 24. The co‐word map retained *n* = 61 keywords from *n* = 9465 author keywords at a minimum occurrence threshold of *n* = 55. These reduced network units were created for interpretive clarity and should not be conflated with the full screened publication corpus.

No eligible foresight‐related nursing publications were identified before 1990, confirming that the effective bibliometric timeline begins in the early 1990s despite the broader exploratory search back to 1970. After database filtering to English‐language journal articles and reviews, records were screened for substantive alignment with future‐oriented framing in nursing contexts, and Delphi/consensus studies were retained only when authors explicitly framed them as future planning, anticipatory role or competency development, or forward‐looking priority setting.

The study‐selection workflow is presented in Supporting Figure S2, which provides a record‐level verification workflow for identification, screening, exclusion, inclusion, and analytical subset construction. The full screening decision trail, including pass/fail variables, evidence fields, and exclusion reasons for each imported record, is provided in Supporting Data File S1.

Publication output increased substantially after 2010, as shown in Supporting Figure S1, which presents annual publication counts for the verified screened corpus. The citation line in Supporting Figure S1 represents the sum of Web of Science Times Cited values for records published in each year; therefore, it should be interpreted as accumulated citation impact by publication cohort, not as citations received within each calendar year. As a descriptive mapping approach, bibliometric analysis cannot determine whether a topic is “emerging” in practice, nor can it forecast future directions; it can only show how published scholarship is distributed, connected, and cited within the retrieved dataset.

### 3.2. Co‐Citation Analysis

Co‐citation analysis was conducted to map frequently co‐cited references and citation‐based groupings within the verified corpus of future‐framed nursing scholarship. This technique identifies how often pairs of references are cited together across publications, allowing the mapping of references that function as citation anchors within the retrieved dataset [15]. From the final verified corpus of *n* = 3137 records, *n* = 97,859 cited references were identified. To maintain interpretability while retaining the most connected intellectual anchors, a minimum citation threshold of *n* = 24 was applied. This threshold retained *n* = 64 cited references for the co‐citation network visualized in VOSviewer. The map continued to show strong consensus‐oriented co‐citation patterns, with central references concentrated around Delphi methodology, consensus reporting, measurement standards, and nursing leadership/education reform.

Within the mapped corpus, Delphi‐based consensus approaches were highly visible as a route through which future‐framed nursing work was operationalized. The most influential documents, including Hasson et al. [22], Keeney et al. [23, 24], McKenna [25], and Powell [26], represent seminal guidance on the design, rigor, and critical appraisal of Delphi studies. Contemporary methodological refinements are reflected in the high link strength of Jünger et al. [14] and Diamond et al. [13], which formalized reporting criteria and consensus standards for Delphi‐based inquiry.

Earlier conceptual contributions, such as Goodman [27] and Jones and Hunter [11], provided foundational discussions on consensus methods in health research. These works established frameworks that remain influential in foresight‐oriented nursing scholarship. Collectively, these co‐cited references indicate that the retrieved corpus is strongly anchored in Delphi methodology, consensus development, and methodological rigor. This pattern suggests that future‐framed nursing work in the indexed dataset has often been operationalized through structured expert judgment, rather than demonstrating that Delphi is equivalent to futures thinking or that it defines the full conceptual range of nursing foresight.

Total link strength values further illustrate the centrality of these documents. Higher link strengths indicate stronger conceptual influence and deeper integration within the scholarly discourse. The top 10 most co‐cited references, presented in Supporting Table S2, represent the core methodological literature that continues to guide foresight‐driven research, agenda setting, and strategic planning in nursing and health systems.

Across the retained co‐citation network, the methodological reference points encompassed statistical power analysis [28], electronic research data capture and informatics infrastructure [29], systematic‐review reporting guidance [30], and content‐validity assessment [31]. Their presence illustrates that the mapped literature draws on broad research‐quality, measurement, data‐management, and reporting frameworks in addition to Delphi and futures‐oriented scholarship. Co‐citation analysis identified four citation‐based clusters within the retained reference set on futures thinking and strategic foresight in nursing. Each cluster reflects a cohesive body of work that is frequently cited together, indicating shared methodological foundations, conceptual orientations, or research applications. The degree of interconnectedness among documents, measured through total link strength, demonstrates how often pairs of studies appear together in reference lists. This metric highlights the extent to which certain works function as conceptual anchors within the field [17, 18].

**FIGURE 1:**
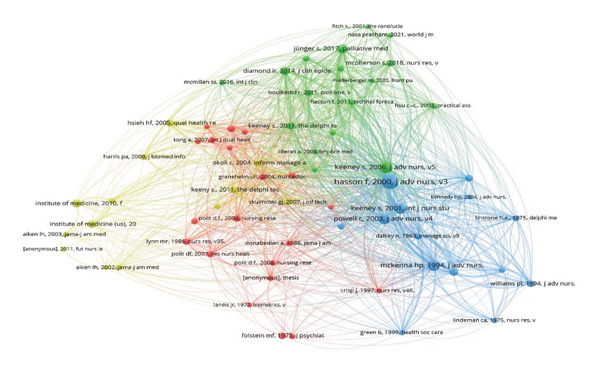
Co‐citation network of retained cited references in future‐framed nursing scholarship. Note: The map visualizes *n* = 64 cited references retained from *n* = 97,859 cited references at a minimum citation threshold of *n* = 24. Node size indicates relative citation prominence; links indicate co‐citation relationships; colors indicate cluster membership.

The resulting co‐citation map (Figure 1) visually depicts these relationships and reveals how foundational studies cluster around core themes such as Delphi methodology, consensus development, methodological rigor, and expert‐based forecasting. Together, these clusters show which methodological and policy‐oriented references were most frequently co‐cited in the retrieved corpus.

• Cluster 1, “Methodological Scaffolding and Evidence Standards in Future‐Framed Nursing Research”: Cluster 1 (20 publications) primarily reflects methodological scaffolding that future‐framed nursing papers use to establish credibility and support translation (e.g., qualitative trustworthiness, measurement/validity conventions, reporting guidance, and implementation frameworks), rather than a distinct foundation of futures theory. Interpreted within the limits of science mapping, this cluster should be read as an interpretive signal: in this indexed corpus, future‐framed claims are most often anchored in familiar nursing research standards for study design, appraisal, reporting, and uptake—not in foundational futures scholarship itself. Aiken et al. [2] co‐occur within this configuration, suggesting that workforce and outcomes evidence is frequently used to strengthen the legitimacy of forward‐looking arguments. Accordingly, this pattern suggests the value of stating explicitly what makes a study futures‐oriented (e.g., scanning logic, scenario reasoning, backcasting steps, or a futures literacy frame) and of connecting that futures logic to established standards, rather than assuming that citing rigorous texts alone communicates a futures orientation [15, 18].
• Cluster 2 (green): This cluster is titled “Delphi‐Based Consensus and Quality Frameworks in Future‐Framed Health and Nursing Research.” This cluster, with 17 publications, is anchored in Delphi methodology and consensus techniques that underpin much of futures‐oriented work in health and nursing. Highly co‐cited contributions by Keeney and colleagues provide practical and conceptual guidance on designing and conducting Delphi studies in nursing and health research [23, 24, 32]. Methodological reviews and reporting standards, such as those by Hsu and Sandford [33], Akins et al. [34], Boulkedid et al. [12], Diamond et al. [13], McMillan et al. [35], and Nasa & Patel [36], converge to define what constitutes rigorous panel selection, iteration, stability of responses, and transparent reporting. Within this cluster, several texts extend Delphi beyond methodology into applied consensus and implementation science. The RAND/UCLA Appropriateness Method connects expert consensus with clinical decision‐making and service design [37], while Donabedian’s classic model of structure, process, and outcomes frames quality as the ultimate anchor for appropriateness and foresight in care delivery [1]. In parallel, Jünger et al. [14], Trevelyan and Robinson [38], Niederberger and Spranger [39], McPherson et al. [40], and von der Gracht [41] illustrate how Delphi has been adapted across palliative care, integrative medicine, public health, nursing measurement, and technology forecasting, emphasizing its flexibility as a tool for shaping future standards and priorities. Within this corpus, the density of co‐citations around Delphi guidance and consensus reporting standards indicates that forward‐looking nursing work (as operationalized in this review) is frequently conducted through structured expert consensus and guideline/indicator development. This finding describes the dominant methodological pathway represented in the retrieved literature; it should not be read as evidence that consensus approaches are expanding in all forms of nursing futures work beyond this indexed dataset.
• Cluster 3 (blue): The cluster, with 14 publications, titled “Conceptual and Historical Foundations of Delphi and Consensus Methods” represents the conceptual and historical roots of Delphi and consensus methods that underpin contemporary future‐framed work in nursing and health research. Early nursing papers by Lindeman [42], Duffield [43], Crisp et al. [44], Williams and Webb [45], and Green et al. [46] demonstrate how Delphi was first used to define roles, competencies, and priorities in nursing practice and education. Foundational critiques and methodological reflections by Goodman [27], McKenna [25], Hasson et al. [22], and Powell [26] examine key issues such as anonymity, rounds of iteration, stability of responses, and what constitutes genuine consensus, offering nuanced guidance on the appropriate use of Delphi in complex professional contexts. Keeney et al. [24] consolidate these developments in a critical review of Delphi as a research methodology in nursing, while Jones and Hunter [11] situate Delphi within a broader family of consensus techniques in health services research. The cluster also links back to classic methodological sources, including Dalkey’s original formulation of Delphi as a systematic forecasting method (Dalkey and Helmer [9] and Linstone’s early monograph on the Delphi technique [10]. In the context of the present review, these works are best understood as part of the historical development of expert‐based consensus and forecasting methods. Interpreted cautiously, this cluster shows how expert judgment became a prominent way of organizing future‐framed role, service, and priority discussions in the retrieved corpus. Although Cluster 3 shares thematic proximity with Cluster 2 through its focus on Delphi and consensus‐building, it represents an earlier and more conceptual layer within the intellectual structure. Cluster 2 captures modern methodological standards and applied consensus frameworks, whereas Cluster 3 reflects the historical, philosophical, and foundational debates that shaped the development of Delphi as an expert‐based consensus and forecasting method.
• Cluster 4 (yellow): The cluster, with 13 publications, titled “Nursing Leadership, Education Reform, and Evidence for Future‐Ready Workforces” links methodological tools with influential visions for the future of nursing. Method papers by Okoli and Pawlowski [47], Skulmoski et al. [48], Hsu and Sandford [33], de Villiers et al. [49], and Hasson et al. [50] describe practical steps for planning, sampling, and analyzing Delphi studies, positioning Delphi as a flexible mechanism for building expert consensus on emerging priorities. Alongside these works, several landmark policy and standards documents articulate long‐term directions for nursing education and leadership. The Essentials of Baccalaureate Education for Professional Nursing Practice [4] and the Institute of Medicine reports The Future of Nursing: Leading Change, Advancing Health [5, 51] outline strategic goals for expanded nursing roles, higher educational preparation, and stronger leadership in transforming health systems. Aiken et al. [3] provide compelling empirical evidence that nurse staffing and education levels are associated with patient outcomes, giving a quantitative backbone to these reform agendas. Interpreted cautiously, this cluster reflects normative and policy‐oriented visions of what nursing workforce, education, and leadership development should build toward within the retrieved corpus. It should not be read as evidence that these publications, in themselves, constitute explicit strategic foresight practice. Delphi methods appear here primarily as tools to operationalize these visions, for example, by reaching consensus on competencies or curricula. The cluster also highlights a gap, since most documents focus on hospital and traditional education settings, pointing to the need for future work that extends these leadership frameworks to digital health, planetary health, and community‐based care.

Supporting Table S3 presents the co‐citation clusters, their thematic focus, number of studies, and key representative publications on Futures Thinking and Strategic Foresight in Nursing.

### 3.3. Co‐Word Analysis

Co‐word analysis was conducted to map the author‐keyword footprint of future‐framed nursing scholarship as expressed through author keywords—showing how “future orientation” is indexed, labeled, and clustered in journal metadata rather than forecasting nursing’s future [15, 17]. Accordingly, the analysis identifies dominant framings and application contexts in the indexed record, not normative priorities or real‐world adoption trajectories.

As shown in Supporting Table S4, high‐frequency domain keywords (e.g., care, nurses, nursing, education) primarily function as contextual anchors—indicating where futures framing is embedded in nursing journals—rather than signaling foresight theory or futures constructs. By contrast, the prominence of Delphi technique and consensus indicates that, in this indexed corpus, future framing is most often operationalized through convergence: translating forward‐looking concerns into competencies, priorities, indicators, and guidelines via structured expert agreement. This is analytically distinct from futures thinking as a plural‐futures orientation, which also requires methods that preserve uncertainty, divergence, and engagement with undesirable or contested futures [7, 8].

The recurring presence of terms such as quality, impact, outcomes, and management further suggests that much of this future‐framed work is oriented toward performance evaluation and improvement, consistent with anticipatory governance and implementation‐facing planning rather than exploratory or speculative futures inquiry. Similarly, keywords such as prevalence, depression, and dementia reflect persistent societal and health‐system pressures that motivate forward‐looking planning but do not in themselves indicate explicit engagement with plural or alternative futures.

**FIGURE 2:**
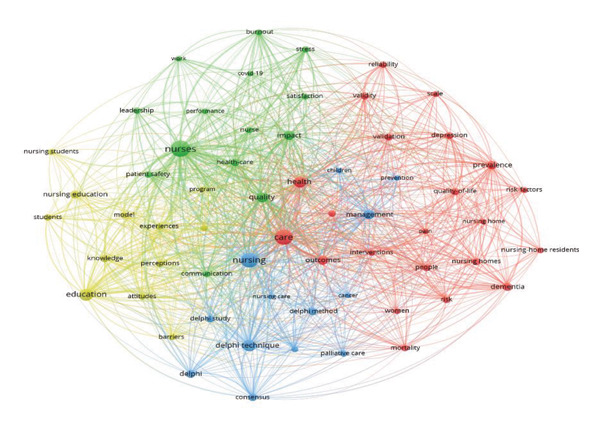
Co‐word network of thematic concentrations in future‐framed nursing scholarship. Note: The map visualizes *n* = 61 author keywords retained from *n* = 9465 keywords at a minimum occurrence threshold of *n* = 55. Node size indicates keyword frequency; links indicate co‐occurrence relationships; colors indicate cluster membership.

Viewed as a descriptive map of the retrieved corpus, the co‐word network (Figure 2) indicates that within journal‐indexed nursing literature, future orientation is most often enacted as applied planning embedded in substantive domains, rather than as an explicit, standalone engagement with futures theory or foresight toolkits [15, 17]. This explains why high‐frequency domain keywords dominate the network: in this dataset, they function primarily as sites where futures framing is enacted, commonly via consensus‐based priority setting, guideline development, and standards work.

Crucially, this pattern substantiates—rather than undermines—the conceptual distinction raised by reviewers between technical operation and plural‐futures engagement. While futures framing is empirically visible across nursing domains, the explicit foresight toolkit footprint (e.g., horizon scanning, scenario development, backcasting, and futures literacy) remains comparatively sparse at the keyword level. As demonstrated in Supporting Table S5, explicit foresight anchor terms occur in only a small proportion of included records, indicating that plural‐futures methods are present but not yet central to the indexed nursing literature [7, 8].

This finding is useful for interpreting the retrieved corpus because it suggests that, within Web of Science metadata, future‐framed nursing work is often indexed through consensus and standards‐related language—convergence‐oriented consensus and standards work—and clarifies where methodological diversification would be required if the field seeks broader engagement with plural, contested, and undesirable futures beyond refinement of preferred standards [7, 8]. As with all science‐mapping outputs, these patterns describe concentrations in indexed metadata and should not be interpreted as forecasts, value judgments, or evidence of adoption without complementary temporal or qualitative analyses [15, 17].

• Cluster 1 (red): This cluster contains 22 keywords labeled “Clinical risk, outcomes, and measurement in care contexts.” Cluster 1 links future‐framed nursing scholarship to clinical risk, health outcomes, long‐term care, dementia, mortality, quality of life, and measurement‐oriented research. The co‐occurrence of care, outcomes, mortality, risk, quality‐of‐life, and prevalence suggests that future‐framed nursing work is frequently embedded in clinical and population‐health concerns where care quality, risk assessment, and outcome monitoring are central. This interpretation is consistent with Donabedian’s structure–process–outcome model of care quality and with nursing outcomes research linking care environments and staffing conditions to patient outcomes [1–3]. The presence of reliability, validity, validation, and scale indicates that part of this literature is indexed through instrument development, psychometric appraisal, and measurement‐oriented work. These terms suggest that future‐framed nursing scholarship is not only concerned with substantive clinical domains but also with how risks, needs, outcomes, and care quality are measured and made visible in research and practice. This measurement emphasis aligns with established nursing research guidance on instrument development, validity, and reliability [52–54]. From a bibliometric perspective, this cluster should be interpreted as a clinical outcomes and measurement concentration, rather than as an explicit foresight‐theory cluster. The presence of dementia, depression, nursing homes, pain, women, and quality‐of‐life indicates that future‐framed language is often embedded in high‐burden clinical and long‐term care contexts. However, these keywords do not by themselves demonstrate explicit engagement with plural‐futures methods such as scenario planning, horizon scanning, or backcasting. As with all co‐word clusters, the pattern should be read as a concentration in author keywords within the retrieved corpus, not as evidence of causality, prediction, or real‐world adoption trends [15–17].
• Cluster 2 (green): This cluster, with 14 keywords labeled “Nursing workforce, quality, leadership, and work environment,” is defined by keywords—burnout, communication, COVID‐19, healthcare, impact, leadership, nurse, nurses, patient safety, performance, quality, satisfaction, stress, and work. This cluster centers on nurses as the workforce through which quality, patient safety, leadership, performance, and organizational conditions are enacted. The co‐occurrence of nurses, quality, patient safety, leadership, performance, and healthcare suggests that future‐framed nursing scholarship is frequently embedded in concerns about how nursing work contributes to safe and effective health‐system performance. This interpretation is consistent with Donabedian’s structure–process–outcome model of quality and with nursing outcomes research showing that staffing, education, and work environments are associated with mortality, burnout, job dissatisfaction, and patient outcomes [1–3]. The presence of burnout, stress, work satisfaction, and COVID‐19 further indicates that future‐framed nursing scholarship also addresses workforce strain, occupational stressors, and system resilience. These terms suggest that the future‐facing literature is not limited to clinical outcomes but also reflects concern with the sustainability of nursing work itself. This aligns with policy and professional calls for stronger nursing leadership, workforce preparation, and health‐system transformation in response to changing population needs and recurrent system disruptions [5, 55]. From a bibliometric perspective, this cluster should be interpreted as a workforce quality and leadership concentration, rather than as evidence that the retrieved literature directly forecasts the future of nursing work. The cluster shows that future‐framed language is frequently attached to quality, safety, leadership, and workforce sustainability concerns, but the keywords themselves do not demonstrate explicit use of plural‐futures tools such as scenarios, scanning, or backcasting. As with the other co‐word clusters, the pattern should be read as a concentration in author keywords within the screened corpus, not as evidence of causality, prediction, or real‐world adoption trends [15–17].
• Cluster 3 (blue): “Delphi/consensus‐based nursing management and guideline development.” This cluster is defined by 13 keywords (cancer, children, consensus, Delphi, Delphi method, Delphi study, Delphi technique, guidelines, management, nursing, nursing care, palliative care, and prevention). The cluster represents the clearest convergence‐oriented methodological concentration in the co‐word map. The grouping of Delphi‐related terms with consensus, guidelines, management, nursing care, palliative care, cancer, children, and prevention indicates that structured expert agreement is frequently used to develop guidance, priorities, standards, and management recommendations in nursing and health‐care contexts. The prominence of Delphi, the Delphi method, Delphi studies, the Delphi technique, and consensus is consistent with the wider methodological literature describing Delphi as a structured approach for expert judgment, consensus development, and decision support in health and nursing research [11, 22–24, 50]. The co‐occurrence of these terms with guidelines and management also aligns with methodological and reporting standards that emphasize transparency in panel selection, iteration, consensus thresholds, and reporting quality in Delphi‐based guideline or indicator development [12–14, 36, 39]. In the context of this review, Cluster 3 supports the manuscript’s central interpretation that future‐framed nursing scholarship is often made publication‐visible through consensus‐based pathways. In the context of this review, Cluster 3 supports the interpretation that future‐framed nursing scholarship is often made publication‐visible through consensus‐based pathways, particularly where expert agreement is used to define priorities, standards, competencies, or guidance. This cluster is therefore best read as evidence of a consensus and guideline‐development pathway within the screened corpus.
• Cluster 4 (yellow): This cluster has 12 keywords labeled “Nursing education, implementation, attitudes, and learning/program development.” Cluster 4 is defined by 12 tightly connected keywords—attitudes, barriers, education, experiences, implementation, knowledge, model, nursing education, nursing students, perceptions, program, and students. This cluster shows that future‐framed nursing scholarship is also concentrated in educational and implementation contexts, particularly where studies examine knowledge, attitudes, perceptions, barriers, student preparation, and program development. Compared with the Delphi‐centered cluster, this cluster is less method‐specific and more focused on educational uptake, learning environments, and the practical translation of future‐oriented priorities into programs or curricula. The prominence of education, nursing education, nursing students, students, knowledge, and programs is consistent with professional and policy frameworks that position nursing education as central to preparing a workforce capable of leadership, innovation, systems transformation, and health equity work [4, 5, 55]. The presence of attitudes, experiences, and perceptions suggests that the literature also attends to how learners, nurses, and stakeholders understand educational or organizational change. These terms align with qualitative and interpretive traditions in nursing research that emphasize experience, meaning, trustworthiness, and contextual understanding [56–60]. The co‐occurrence of implementation, barriers, model, and program indicates that this cluster also reflects concern with translating educational or practice innovations into usable forms. This interpretation is consistent with implementation‐science perspectives that emphasize context, barriers, intervention characteristics, and process when moving evidence or programs into practice [61]. From a bibliometric perspective, Cluster 4 suggests that nursing education remains an important site for embedding future‐oriented competencies and programmatic change. However, the relative absence of explicit foresight‐toolkit terms within this cluster indicates that these educational discussions are often indexed through general education and implementation language rather than through explicit futures methods such as scenario planning, horizon scanning, backcasting, or futures literacy [7, 8].

Supporting Table S6 presents four‐co‐word clusters that summarize the main conceptual concentrations identified in the indexed literature on future‐framed nursing scholarship. These clusters describe how keywords are grouped in the retrieved dataset; they should not be interpreted as evidence of causal relationships, real‐world adoption, or the overall evolution of discipline.

### 3.4. Discussion and Interpretive Implications

This science‐mapping review describes publication‐visible patterns in future‐framed nursing scholarship. Within the verified Web of Science corpus, future‐framed work is most often represented through consensus‐oriented pathways, including Delphi guidance, consensus measurement, reporting standards, competencies, indicators, and guidelines, as reflected in the co‐citation and co‐word maps. Explicit foresight tools, including scanning, scenario development, backcasting, and futures literacy, remain comparatively less visible in titles, abstracts, and author keywords [7, 8].

These findings should be interpreted as bibliometric patterns within the screened corpus, not as causal explanations, forecasts, or definitive indicators of the discipline’s future direction. The maps show how future‐framed nursing work becomes visible in journal metadata and citation networks, consistent with the descriptive purpose and limits of co‐citation and co‐word science mapping [15–18].

Because the sequencing figure is heuristic rather than a direct bibliometric output, it has been moved to the supporting information as Supporting Figure S3. The main discussion, therefore, focuses on the publication‐visible patterns identified in the co‐citation and co‐word maps.

### 3.5. Implications for Research and Scholarship

This mapping is descriptive and interpretive: it identifies a dominant publication‐visible pattern in the retrieved journal literature and clarifies where explicit foresight toolkits are comparatively underrepresented.

Across the four co‐word clusters, future‐framed language most commonly co‐occurred with themes related to (1) clinical risk, outcomes, long‐term care, and measurement; (2) nursing workforce, quality, leadership, patient safety, and work environment; (3) Delphi/consensus‐based nursing management and guideline development; and (4) nursing education, implementation, attitudes, and learning/program development. These clusters should be interpreted as dominant thematic concentrations within the retrieved dataset and as an index of how “future orientation” is being framed in nursing‐indexed journal articles. They are not causal explanations, predictions, or definitive drivers of nursing’s future; they are descriptive outputs of keyword and citation relationships shaped by database coverage, indexing practices, and the study’s operational definition of futures orientation [15–17].

At the keyword level, explicit strategic foresight toolkits, such as horizon scanning, scenario development, and futures literacy frameworks, were comparatively less visible than consensus‐based approaches, suggesting that the published nursing literature captured in Web of Science has more frequently operationalized forward‐looking work through structured expert agreement than through a broader portfolio of foresight methods [7, 8]. Accordingly, future studies may benefit from three reporting and design refinements: (1) stating an explicit futures/foresight definition early, including whether it is used as an orientation or method; (2) reporting how futures orientation was established, including anchor terms and study purpose; and (3) clarifying when Delphi is being used for convergence rather than being treated implicitly as a proxy for foresight.

### 3.6. Descriptive Interpretation of Co‐Word Clusters

The four co‐word clusters show where future‐framed nursing scholarship is most visible in author keywords within the verified Web of Science corpus. These clusters are descriptive keyword concentrations, not causal explanations, forecasts, or indicators of real‐world adoption. They should therefore be interpreted as metadata‐based signals of how future‐oriented nursing topics are indexed and grouped in the retrieved literature, consistent with the descriptive purpose of co‐word analysis [15–17].

#### 3.6.1. Clinical Risk, Outcomes, and Measurement

Cluster 1 shows that future‐framed nursing scholarship is frequently indexed through clinical risk, outcomes, long‐term care, dementia, quality of life, and measurement‐related terms. This suggests that future‐oriented nursing work is often linked to the assessment and monitoring of care needs rather than to explicit foresight theory. This interpretation is consistent with quality‐of‐care and nursing outcomes research linking care structures, processes, staffing, and work environments to patient outcomes [1–3]. The presence of reliability, validity, validation, and scale also suggests that measurement and instrument development work are important parts of this keyword concentration [62, 63].

#### 3.6.2. Workforce, Quality, and Leadership

Cluster 2 links future‐framed scholarship with nurses, quality, patient safety, leadership, performance, stress, burnout, satisfaction, work, and COVID‐19. This indicates that workforce sustainability and quality/safety concerns are major publication‐visible sites where future‐oriented nursing questions appear. These patterns align with research connecting nurse staffing, work environment, burnout, and patient outcomes, as well as policy calls for stronger nursing leadership and workforce preparation in transforming health systems [2, 3, 5, 55].

#### 3.6.3. Consensus‐Based Guideline and Management Development

Cluster 3 shows strong clustering around Delphi, consensus, guidelines, management, nursing care, palliative care, prevention, cancer, and children. This supports the interpretation that structured expert consensus is a prominent publication‐visible pathway for translating future‐oriented concerns into standards, priorities, competencies, and guidance. This pattern is consistent with established uses of Delphi and consensus methods in health and nursing research, including expert panel selection, iterative rating, consensus criteria, and reporting standards [11–14, 22–24, 64, 65].

#### 3.6.4. Education and Implementation

Cluster 4 links education, nursing education, nursing students, knowledge, attitudes, perceptions, barriers, program development, and implementation. This suggests that future‐oriented nursing scholarship is also embedded in educational preparation and implementation processes, although explicit foresight tools remain less visible in author keywords. The educational emphasis aligns with professional and policy frameworks that position nursing education as central to leadership, innovation, health equity, and system transformation [4, 5, 55]. The implementation‐related terms also align with implementation science perspectives emphasizing context, barriers, intervention characteristics, and process in translating evidence or programs into practice [61].

### 3.7. Research Development and Equity

Future research should extend this bibliometric map through full‐text review, cross‐database replication, and gray‐literature triangulation to test whether the publication‐visible patterns identified in Web of Science remain stable across other databases and evidence sources [15, 16, 18, 64]. In particular, studies should compare explicitly foresight‐labeled work with future‐framed consensus studies, examine regional visibility and Global South representation, and assess whether the consensus‐dominant pattern observed in Web of Science is also evident in Scopus, CINAHL, PubMed, policy documents, and other policy‐facing sources [8, 55].

## 4. Conclusion, Limitations, and Future Research

This science‐mapping review screened 4478 raw Web of Science records and synthesized a final verified corpus of 3137 future‐framed nursing records published between 1990 and 2025. Within this screened corpus, the indexed journal record shows a clear publication‐visible pattern: future‐framed nursing work is most often represented through consensus‐oriented pathways (Delphi guidance, consensus measurement, and reporting standards), while explicit foresight toolkits (scanning, scenarios, backcasting, and futures literacy) remain comparatively sparse in titles/abstracts/keywords. Co‐word themes concentrate on (1) clinical risk, outcomes, long‐term care, and measurement; (2) nursing workforce, quality, leadership, patient safety, and work environment; (3) Delphi/consensus‐based nursing management and guideline development; and (4) nursing education, implementation, attitudes, and learning/program development. Future studies should report more clearly how futures or foresight logic is used, especially when consensus methods are applied to future‐oriented priorities, competencies, standards, or guidance.

Several limitations should be acknowledged. First, the corpus is restricted to English‐language journal articles and reviews indexed in Web of Science Core Collection. The findings therefore reflect only the nursing‐related scientific production captured in that database and should not be interpreted as a comprehensive representation of all future‐framed nursing scholarship. Relevant scholarship may be underrepresented if indexed elsewhere, including Scopus, CINAHL, PubMed, regional databases, gray literature, policy documents, and non‐English sources. This is especially important for applied foresight and policy‐facing work, which is often disseminated outside conventional journal indexing pathways. In addition, metadata‐visible retrieval and screening may miss futures‐oriented work that is implicit in full text rather than explicit in titles, abstracts, or keywords. Although the record‐level audit improves transparency and reproducibility, screening remained dependent on metadata‐visible evidence rather than full‐text review of every record. Some future‐framed nursing studies may therefore have been missed if future orientation was implicit in the full text but not visible in titles, abstracts, keywords, source fields, or Web of Science categories. The science‐mapping outputs are descriptive and parameter‐sensitive, not causal or predictive. The co‐citation map visualizes *n* = 64 cited references retained from *n* = 97,859 cited references at a citation threshold of *n* = 24, and the co‐word map visualizes *n* = 61 keywords retained from *n* = 9465 author keywords at an occurrence threshold of *n* = 55. These reduced analytical subsets improve visual interpretability but do not reproduce the full corpus in raw form. Because the threshold values were selected pragmatically for interpretability, the resulting cluster structures should be interpreted as informative visual mappings rather than fixed or exhaustively validated representations of the field. To strengthen transparency, we conducted a threshold‐proximity sensitivity check by comparing the final co‐citation and co‐word thresholds with adjacent lower and higher threshold settings. This check suggested that the broad intellectual and thematic interpretations were stable, although exact node inclusion, cluster boundaries, and visual density remained parameter‐sensitive. Formal cluster‐stability validation and cross‐database triangulation were not undertaken and should be prioritized in future work.

Future research should broaden dataset comprehensiveness by replicating the analysis across Scopus, CINAHL, and PubMed with formal stability checks and by triangulating journal‐indexed findings with gray literature and policy documents, where applied foresight often occurs. Such work should compare whether the consensus‐dominant pattern observed in Web of Science remains stable across databases, indexing systems, and gray literature sources. Additional work should also analyze foresight‐labeled studies versus future‐framed consensus studies as distinct streams, use text‐mining or full‐text approaches to detect implicit foresight practices, and expand equity‐focused analyses of regional visibility and risk contexts. Future empirical work could examine, beyond bibliometric metadata, how different combinations of futures‐oriented and consensus‐oriented methods are used in nursing planning, education, and workforce research.

## Funding

No funding was received for this manuscript.

## Conflicts of Interest

The authors declare no conflicts of interest.

## Supporting Information

Additional supporting information can be found online in the Supporting Information section.

## Supporting information


**Supporting Information** The supporting information for this article is provided as separate files. It includes Supporting Table S1, which presents the database search string and its justification; Supporting Table S2, showing the top 10 documents with the highest co‐citation counts and total link strength; Supporting Table S3, summarizing the co‐citation clusters on futures thinking and strategic foresight in nursing; Supporting Table S4, listing the top 15 keywords in the co‐occurrence analysis; Supporting Table S5, presenting the occurrence of futures/foresight anchor terms in the included records; Supporting Table S6, describing the co‐word clusters; and Supporting Table S7, presenting a threshold‐proximity sensitivity check for the co‐citation and co‐word maps. The Supporting figures include Supporting Figure S1, which shows annual publication counts and accumulated citation impact by publication year for the verified screened corpus; Supporting Figure S2, which presents the record‐level verification workflow for screening the Web of Science bibliometric corpus; and Supporting Figure S3, which provides a heuristic illustration of possible relationships between divergence‐oriented futures methods and later‐stage consensus tools. In addition, we provide Supporting Data File S1, an Excel screening audit trail documenting all imported records, screening decisions, exclusion reasons, and retained records. This audit file supports the transparency, traceability, and reproducibility of the final screened corpus.

## Data Availability

The data that support the findings of this study are available from the corresponding author upon reasonable request.
