# Supplementary material for: Consensus‐Dominant Futures in Nursing: A Co‐Citation and Co‐Word Science‐Mapping Analysis of Future‐Framed Scholarship (1990–2025)
Source: Nurs Res Pract. 2026 Aug 13;2026:1839511. doi: 10.1155/nrp/1839511 (PMC13473835; doi:10.1155/nrp/1839511)
Supplement: Supplementary file 1 [file NRP-2026-1839511-s001.zip › Supplementary Tables and Figure.docx]

**Supplementary Table S1: Search string used for database search**

| Keyword | Justification |
| --- | --- |
| TS=(nurs)* | Ensures retrieval of nursing-related literature. The wildcard * captures variations such as nurse, nurses, nursing, and related terms across topics. |
| AND TS=( "futures thinking" OR "strategic foresight" OR "foresight planning" OR "foresight" OR "scenario planning" OR "scenario analysis" OR scenario* OR "horizon scanning" OR "environmental scanning" OR scanning OR "futures literacy" OR "anticipatory governance" OR backcasting OR visioning OR "three horizons" OR "futures triangle" OR "future of nursing" OR "future studies" OR "futures studies" OR trend* OR megatrend* ) | Captures futures/foresight constructs and future-oriented analytical approaches commonly used in nursing research and policy planning, including strategic foresight toolkits (e.g., scenario planning/analysis, horizon/environmental scanning, backcasting, futures literacy). Delphi and other consensus methods were not used as retrieval terms because they are not synonymous with futures thinking; instead, Delphi/consensus studies were considered at the screening stage only when authors explicitly framed them as future-oriented (e.g., future planning, anticipatory competency/role development, or forward-looking priority setting). |
| AND  TS=("systems thinking" OR "complex adaptive system"* OR "systems approach"* OR "systems mapping")** | Added as a contextual qualifier to capture futures/foresight papers that explicitly use systems/complexity framing. These terms are not treated as futures proxies and are included only alongside the futures/foresight anchor block, so they cannot retrieve general systems-thinking nursing papers on their own. |

**Supplementary Table S2: Top 10 documents with the highest co-citation and total link strength**

| **No** | **Documents** | **Citation** | **Total link strength** |
| --- | --- | --- | --- |
| 1 | Hasson, F. (2000). Research guidelines for the Delphi survey technique. Journal of Advanced Nursing, 32(4), 1008. <https://doi.org/10.1046/j.1365-2648.2000.01567.x> | 286 | 826 |
| 2 | Keeney, S., Hasson, F., & McKenna, H. (2006). Consulting the oracle: Ten lessons from using the Delphi technique in nursing research. Journal of Advanced Nursing, 53(2), 205–212. <https://doi.org/10.1111/j.1365-2648.2006.03716.x> | 167 | 503 |
| 3 | McKenna, H. P. (1994). The Delphi technique: A worthwhile research approach for nursing? Journal of Advanced Nursing, 19(6), 1221–1225. <https://doi.org/10.1111/j.1365-2648.1994.tb01207.x> | 129 | 451 |
| 4 | Keeney, S., Hasson, F., & McKenna, H. P. (2001). A critical review of the Delphi technique as a research methodology for nursing. International Journal of Nursing Studies, 38(2), 195–200. <https://doi.org/10.1016/S0020-7489(00)00044-4> | 117 | 404 |
| 5 | Powell, C. (2003). The Delphi technique: Myths and realities. Journal of Advanced Nursing, 41(4), 376–382. <https://doi.org/10.1046/j.1365-2648.2003.02537.x> | 127 | 402 |
| 6 | Jünger, S., Payne, S. A., Brine, J., Radbruch, L., & Brearley, S. G. (2017). Guidance on conducting and reporting Delphi studies (Delphi consensus): Recommendations based on a methodological review. Palliative Medicine, 31(8), 684–706. <https://doi.org/10.1177/0269216317690685> | 121 | 315 |
| 7 | Diamond, I. R., Grant, R. C., Feldman, B. M., Pencharz, P. B., Ling, S. C., Moore, A. M., & Wales, P. W. (2014). Defining consensus: A systematic review recommends methodologic criteria for reporting of Delphi studies. Journal of Clinical Epidemiology, 67(4), 401–409. <https://doi.org/10.1016/j.jclinepi.2013.12.002> | 99 | 288 |
| 8 | Goodman, C. M. (1987). The Delphi technique: A critique. Journal of Advanced Nursing, 12(6), 729–734. <https://doi.org/10.1111/j.1365-2648.1987.tb01376.x> | 69 | 266 |
| 9 | Jones, J., & Hunter, D. (1995). Qualitative Research: Consensus methods for medical and health services research. BMJ, 311(7001), 376–380. <https://doi.org/10.1136/bmj.311.7001.376> | 86 | 226 |
| 10 | Okoli, C., & Pawlowski, S. D. (2004). The Delphi method as a research tool: An example, design considerations and applications. Information & Management, 42(1), 15–29. <https://doi.org/10.1016/j.im.2003.11.002> | 64 | 207 |

**Supplementary Table S3. Co-citation on Futures Thinking and Strategic Foresight in Nursing**

| **Cluster** | **Cluster label** | **Number of articles** | **Representative publications** |
| --- | --- | --- | --- |
| 1 (red) | Methodological scaffolding, qualitative rigor, measurement, and evidence standards | 20 | Elo & Kyngäs (2008); Graneheim & Lundman (2004); Hsieh & Shannon (2005); Lincoln & Guba (1985); Braun & Clarke (2006); Terry et al. (2017); Polit & Beck (2004; 2006; 2007; 2008); Lynn (1986); Landis & Koch (1977); Cohen (1988); Folstein et al. (1975); Damschroder et al. (2009); Liberati et al. (2009); Tong et al. (2007); Harris et al. (2009); Aiken et al. (2002) |
| 2 (green) | Contemporary Delphi reporting, consensus standards, and methodological quality | 17 | Keeney et al. (2006); Keeney (2010); Keeney (2011); Hsu & Sandford (2007); Akins et al. (2005); Boulkedid et al. (2011); Diamond et al. (2014); McMillan et al. (2016); Nasa & Patel (2021); Fitch (2001); Donabedian (1988); Jünger et al. (2017); Trevelyan & Robinson (2015); Niederberger & Spranger (2020); McPherson et al. (2018); von der Gracht (2012). |
| 3 (blue) | Historical and conceptual foundations of Delphi and expert consensus | 14 | Lindeman (1975); Duffield (1993); Crisp (1997); Williams (1994); Green (1999); Goodman (1987); McKenna (1994); Hasson et al. (2000); Powell (2003); Keeney et al. (2001); Jones & Hunter (1995); Dalkey (1963); Linstone (1975). |
| 4 (yellow) | Nursing leadership, education reform, and future-ready workforce evidence | 13 | Okoli & Pawlowski (2004); Skulmoski et al. (2007); Hsu & Sandford (2007); de Villiers et al. (2005); Keeney (2011); AACN (2008); Institute of Medicine (2010); Institute of Medicine (2011); Institute of Medicine (US) (2011); Aiken et al. (2003). |

**Supplementary Table S4. Top 15 Keywords in the Co-occurrence Analysis**

| **Ranking** | **Keyword** | **Occurrences** | **Total link strength** |
| --- | --- | --- | --- |
| 1. | Care | 531 | 1169 |
| 2. | Nurses | 493 | 1121 |
| 3. | Nursing | 408 | 866 |
| 4. | Education | 310 | 703 |
| 5. | Health | 300 | 688 |
| 6. | delphi technique | 307 | 645 |
| 7. | management | 260 | 639 |
| 8. | Impact | 231 | 561 |
| 9. | Quality | 238 | 558 |
| 10. | Outcomes | 221 | 538 |
| 11. | Prevalence | 200 | 482 |
| 12. | Consensus | 146 | 381 |
| 13. | People | 124 | 363 |
| 14. | Depression | 126 | 347 |
| 15. | Dementia | 136 | 335 |

**Supplementary Table S5. Occurrence of futures/foresight anchor terms in included records (title/abstract/keywords)**

| **Anchor term** | **Occurrence (n)** | **% of included corpus** |
| --- | --- | --- |
| scenario planning / scenario analysis | 43 | 1.37% |
| horizon scanning / environmental scanning | 10 | 0.32% |
| strategic foresight / foresight | 4 | 0.13% |
| futures thinking / futures studies | 7 | 0.22% |
| futures literacy | 0 | 0.00% |
| backcasting | 0 | 0.00% |
| anticipatory governance | 0 | 0.00% |
| three horizons | 0 | 0.00% |
| futures triangle | 0 | 0.00% |

**Supplementary Table S6. Co-word clusters in future-framed nursing scholarship**

| **Cluster No and colour** | **Cluster label** | **Number of keywords** | **Representative Keywords** |
| --- | --- | --- | --- |
| 1 (red) | Clinical risk, outcomes, and measurement in care contexts | 22 | care, health, outcomes, prevalence, dementia, depression, risk, mortality, validation, validity, reliability, scale |
| 2 (green) | Nursing workforce, quality, leadership, and work environment | 14 | nurses, quality, impact, health-care, stress, burnout, leadership, patient safety, communication, satisfaction, work, COVID-19 |
| 3 (blue) | Delphi/consensus-based nursing management and guideline development | 13 | nursing, Delphi technique, management, Delphi, Delphi method, consensus, guidelines, palliative care, cancer, children, prevention |
| 4 (yellow) | Nursing education, implementation, attitudes, and learning/program development | 12 | education, nursing education, knowledge, perceptions, model, experiences, attitudes, students, implementation, barriers, program, nursing students |

**Supplementary Table S7.** Threshold-proximity sensitivity check for co-citation and co-word maps

| **Analysis** | **Minimum threshold tested** | **Total source units** | **Retained units meeting threshold** | **Resolution** | **Minimum cluster size** | **Number of clusters** | **Cluster-size distribution** |
| --- | --- | --- | --- | --- | --- | --- | --- |
| Co-citation analysis | 24 citations | 97,859 cited references | 64 cited references | 0.90 | 1 | 4 | 20, 17, 14, 13 |
| Co-citation analysis | 23 citations | 97,859 cited references | 70 cited references | 0.90 | 1 | 5 | 19, 15, 15, 13, 8 |
| Co-citation analysis | 25 citations | 97,859 cited references | 58 cited references | 0.90 | 1 | 4 | 19, 13, 13, 13 |
| Co-word analysis | 55 occurrences | 9,465 author keywords | 61 keywords | 1.00 | 6 | 4 | 22, 14, 13, 12 |
| Co-word analysis | 54 occurrences | 9,465 author keywords | 63 keywords | 1.00 | 6 | 4 | 19, 16, 15, 13 |
| Co-word analysis | 56 occurrences | 9,465 author keywords | 61 keywords | 1.00 | 6 | 4 | 22, 14, 13, 12 |

Supplementary Figure S1. Annual publications and accumulated citations by publication year in the verified screened corpus.

Note: The figure summarizes n = 3,137 verified eligible records. Bars indicate the number of publications by publication year, and the line indicates the sum of Web of Science Times Cited values for records published in each year. Citation values therefore represent accumulated citations by publication cohort, not citations received within each calendar year.

Supplementary Figure S2. Verification screening workflow for Web of Science bibliometric corpus construction

Note: The figure distinguishes the raw imported Web of Science records (n = 4,478), sequential screening exclusions, and the final verified eligible corpus (n = 3,137). The workflow is supported by the record-level Excel screening audit trail provided in Supplementary Data File S1.

**Supplementary Figure S3**. Heuristic illustration of possible relationships between divergence-oriented futures methods and later-stage consensus tools.

Note. This figure is provided only as an interpretive aid. It is not a bibliometric output, validated framework, or recommended methodological sequence. It illustrates one possible relationship between divergence-oriented futures methods and later convergence-oriented consensus processes when standards, competencies, or guidelines are required.
